# Supplementary material for: Taxonomic Evaluation of the Heyndrickxia (Basonym Bacillus) sporothermodurans Group (H. sporothermodurans, H. vini, H. oleronia) Based on Whole Genome Sequences
Source: Microorganisms. 2021 Jan 26;9(2):246. doi: 10.3390/microorganisms9020246 (PMC7911792; doi:10.3390/microorganisms9020246)
Supplement: Supplementary file 1 [file microorganisms-09-00246-s001.zip › MDPI style Sporo Genomes_210112 Supplemtary file.docx]

Supplementary Table 1 The genome’s core and accessory genes of the assembled contigs were generated by annotation with Prokka v1.14.5 and the pangenome pipeline Roary v3.11. *Results if the Protein Identity interval set to 99% (95% is default).

| All *H. sporothermodurans* (n=36) | | |
| --- | --- | --- |
| Core genes | (99% <= strains <= 100%) | 1837 |
| Soft core genes | (95% <= strains < 99%) | 490 |
| Shell genes | (15% <= strains < 95%) | 1923 |
| Cloud genes | (0% <= strains < 15%) | 2797 |
| Total genes | (0% <= strains <= 100%) | 7047 |
|  | | |
| *H. sporothermodurans* *type strain group* (n=34) | | |
| Core genes | (99% <= strains <= 100%) | 2187 |
| Soft core genes | (95% <= strains < 99%) | 387 |
| Shell genes | (15% <= strains < 95%) | 1669 |
| Cloud genes | (0% <= strains < 15%) | 1406 |
| Total genes | (0% <= strains <= 100%) | 5649 |
|  | | |
| *H. sporothermodurans* (n=36) + *H. oleronia* (n=1) | | |
| Core genes | (99% <= strains <= 100%) | 158 (44)* |
| Soft core genes | (95% <= strains < 99%) | 1697 |
| Shell genes | (15% <= strains < 95%) | 2399 |
| Cloud genes | (0% <= strains < 15%) | 8043 |
| Total genes | (0% <= strains <= 100%) | 12297 |
|  | | |
| *H. sporothermodurans* (n=36) + *H. vini* (n=1) | | |
| Core genes | (99% <= strains <= 100%) | 964 |
| Soft core genes | (95% <= strains < 99%) | 1025 |
| Shell genes | (15% <= strains < 95%) | 2271 |
| Cloud genes | (0% <= strains < 15%) | 5676 |
| Total genes | (0% <= strains <= 100%) | 9936 |
|  | | |
| *H. sporothermodurans* (n=36) + *H. vini* (n=1) + *H. oleronia* (n=1) | | |
| Core genes | (99% <= strains <= 100%) | 164 (42)* |
| Soft core genes | (95% <= strains < 99%) | 819 |
| Shell genes | (15% <= strains < 95%) | 3281 |
| Cloud genes | (0% <= strains < 15%) | 10602 |
| Total genes | (0% <= strains <= 100%) | 14866 |
|  | | |
| *H. oleronia* (n=1) + *H. vini* (n=1) | | |
| Core genes | (99% <= strains <= 100%) | 190 |
| Soft core genes | (95% <= strains < 99%) | 0 |
| Shell genes | (15% <= strains < 95%) | 8979 |
| Cloud genes | (0% <= strains < 15%) | 0 |
| Total genes | (0% <= strains <= 100%) | 9169 |


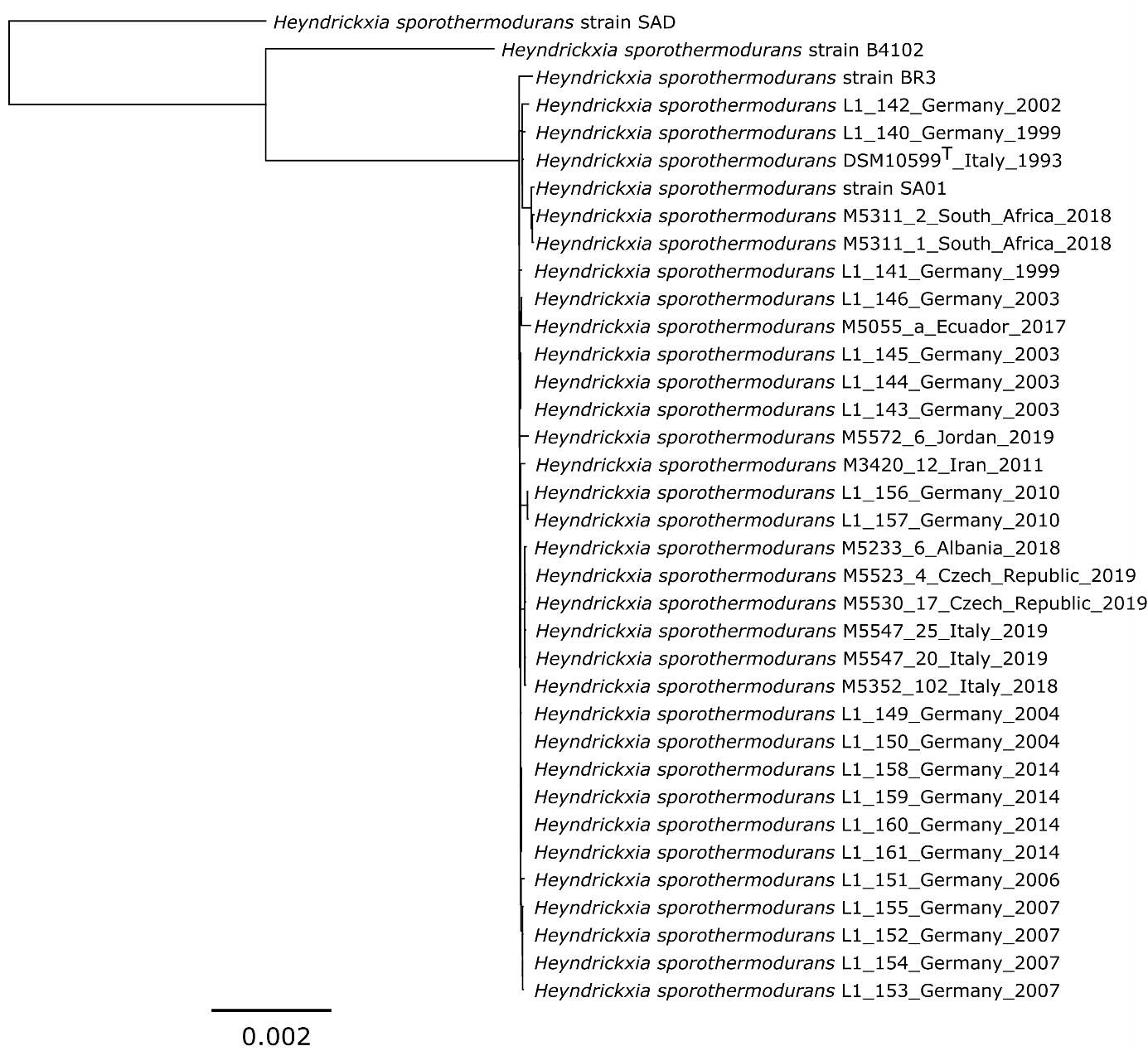


Supplementary Figure 1 Phylogenetic tree analysis of 36 *H. sporothermodurans* strains based on core genes. Codon Tree method selects 1000 single-copy genes (PATRIC PGFams) and analyzes aligned proteins and coding DNA using the program RAxML version 8.2.11, tree was rooted to strain SAD. The branches are scaled in terms of the expected number of substitutions per site.


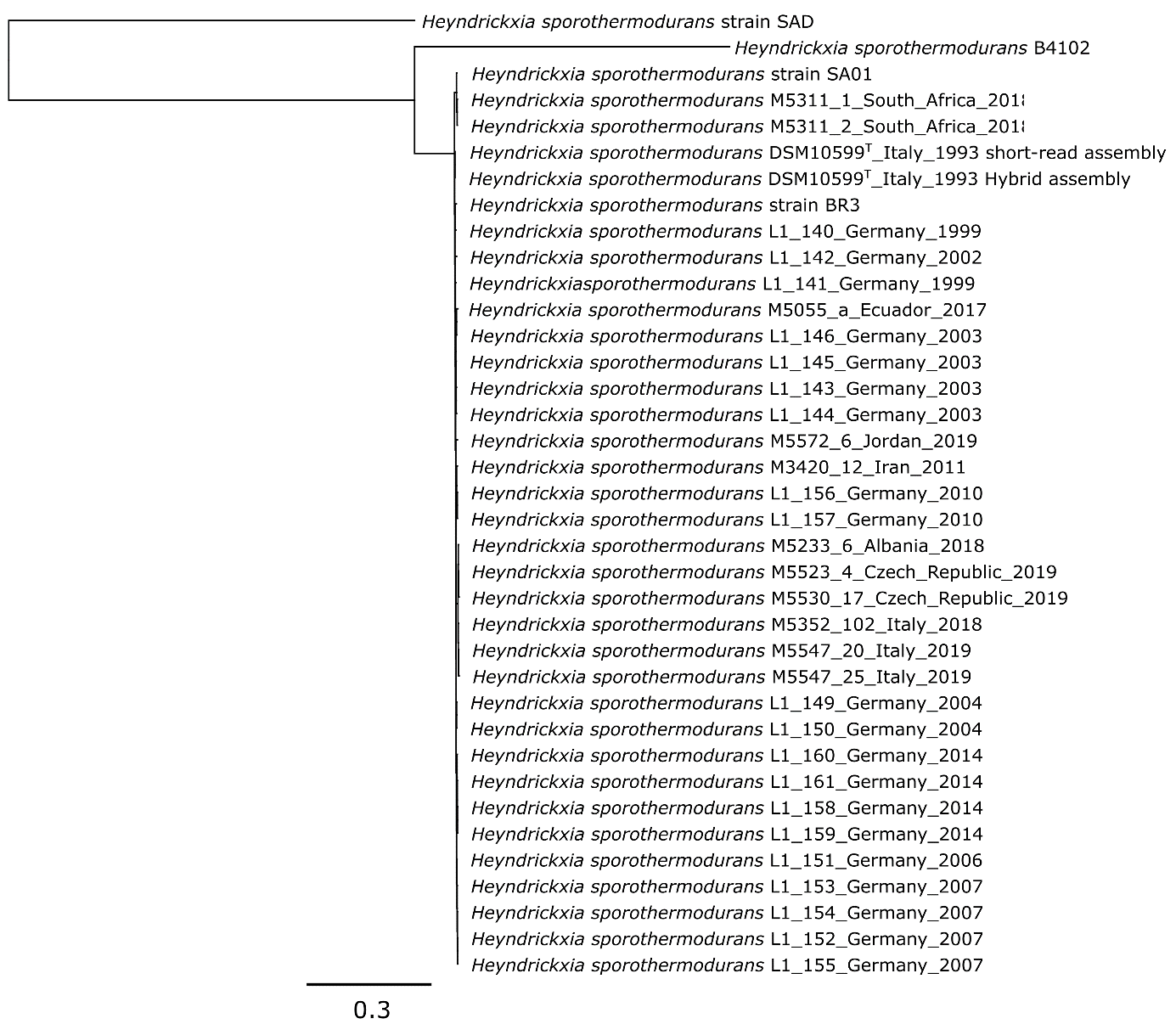


Supplementary Figure 2 Phylogenetic tree analysis of 36 *H. sporothermodurans* strains based on single-nucleotide polymorphisms (SNPs). SNPs were called against the type strain as reference at the CSI Phylogeny 1.4 service. The Newick file was processed with Geneious® and InkScape v0.92. The tree was rooted to strain SAD (based on the single-nucleotide polymorphisms (SNPs). The branches are scaled in terms of the expected number of substitutions per site.

Supplementary Table 2 Overview about the genome-to-genome calculations and the number of SNPs from all strains and species, used in this study. *Initially identified as *H. vini* by 16S. N/D not done.

|  | #ANIb and aligned [] percentage | #DDH and Probability of same species [] percentage | #Number of single nucleotide polymorphisms (SNPs) |
| --- | --- | --- | --- |
| *Heyndrickxia sporothermodurans* strain | Reference: *H. sporothermodurans* (DSM 10599^T^) hybrid assembly | | |
| M3420-12 | 99.86 [92.67] | 99.50 [98.17] | 53 |
| M5055-A | 99.84 [93.26] | 99.60 [98.19] | 75 |
| M5233-6 | 99.89 [93.37] | 99.60 [98.19] | 80 |
| M5311-1 | 99.90 [93.93] | 99.70 [98.21] | 86 |
| M5311-2 | 99.88 [94.36] | 99.70 [98.21] | 81 |
| M5523-4 | 99.85 [93.32] | 99.50 [98.17] | 85 |
| M5530-17 | 99.86 [93.25] | 99.50 [98.18] | 98 |
| M5547-20 | 99.86 [93.49] | 99.60 [98.19] | 86 |
| M5547-25 | 99.88 [93.59] | 99.50 [98.18] | 93 |
| M5352-102 * | 99.96 [94.91] | 99.60 [98.19] | 84 |
| M5572-6 * | 99.86 [93.60] | 99.50 [98.18] | 62 |
| L1_140 | 99.83 [94.39] | 99.70 [98.21] | 29 |
| L1_141 | 99.85 [94.36] | 99.70 [98.22] | 26 |
| L1_142 | 99.87 [94.24] | 99.70 [98.22] | 46 |
| L1_143 | 99.84 [94.40] | 99.70 [98.21] | 31 |
| L1_144 | 99.86 [94.66] | 99.70 [98.21] | 34 |
| L1_145 | 99.86 [94.40] | 99.70 [98.21] | 36 |
| L1_146 | 99.86 [94.38] | 99.70 [98.22] | 47 |
| L1_149 | 99.88 [94.50] | 99.70 [98.21] | 39 |
| L1_150 | 99.88 [94.35] | 99.50 [98.18] | 37 |
| L1_151 | 99.87 [94.15] | 99.60 [98.18] | 43 |
| L1_152 | 99.87 [94.76] | 99.50 [98.18] | 49 |
| L1_153 | 99.89 [94.57] | 99.50 [98.16] | 48 |
| L1_154 | 99.87 [94.45] | 99.50 [98.18] | 52 |
| L1_155 | 99.87 [94.78] | 99.60 [98.2] | 50 |
| L1_156 | 99.87 [93.17] | 99.60 [98.2] | 51 |
| L1_157 | 99.85 [92.78] | 99.60 [98.2] | 53 |
| L1_158 | 99.88 [95.44] | 99.60 [98.18] | 49 |
| L1_159 | 99.86 [95.24] | 99.60 [98.18] | 47 |
| L1_160 | 99.94 [96.17] | 99.60 [98.2] | 49 |
| L1_161 | 99.88 [95.13] | 99.60 [98.19] | 46 |
| *Heyndrickxia sporothermodurans* B4102_Netherlands_2012 | 98.38 [79.32] | 90.40 [95.92] | 4931 |
| *Heyndrickxia sporothermodurans* BR3_Brazil_2012 | 99.75 [87.65] | 99.20 [98.11] | 42 |
| *Heyndrickxia sporothermodurans* SA01_South_Africa_2015 | 99.86 [94.50] | 99.50 [98.17] | 71 |
| *Heyndrickxia sporothermodurans* SAD_South_Africa_2015 | 97.70 [80.38] | 82.00 [92.01] | 7890 |
|  | | | |
| Species | Reference: *H. sporothermodurans* (DSM 10599^T^) hybrid assembly | | |
| *Heyndrickxia vini* LAM0415^T^/ JCM 19841^T^ | 88.97 [64.77] | 39.70 [2.55] | N/D |
| *Heyndrickxia oleronia* DSM 9356^T^ | 76.00 [41.01] | 22.10 [0] | N/D |


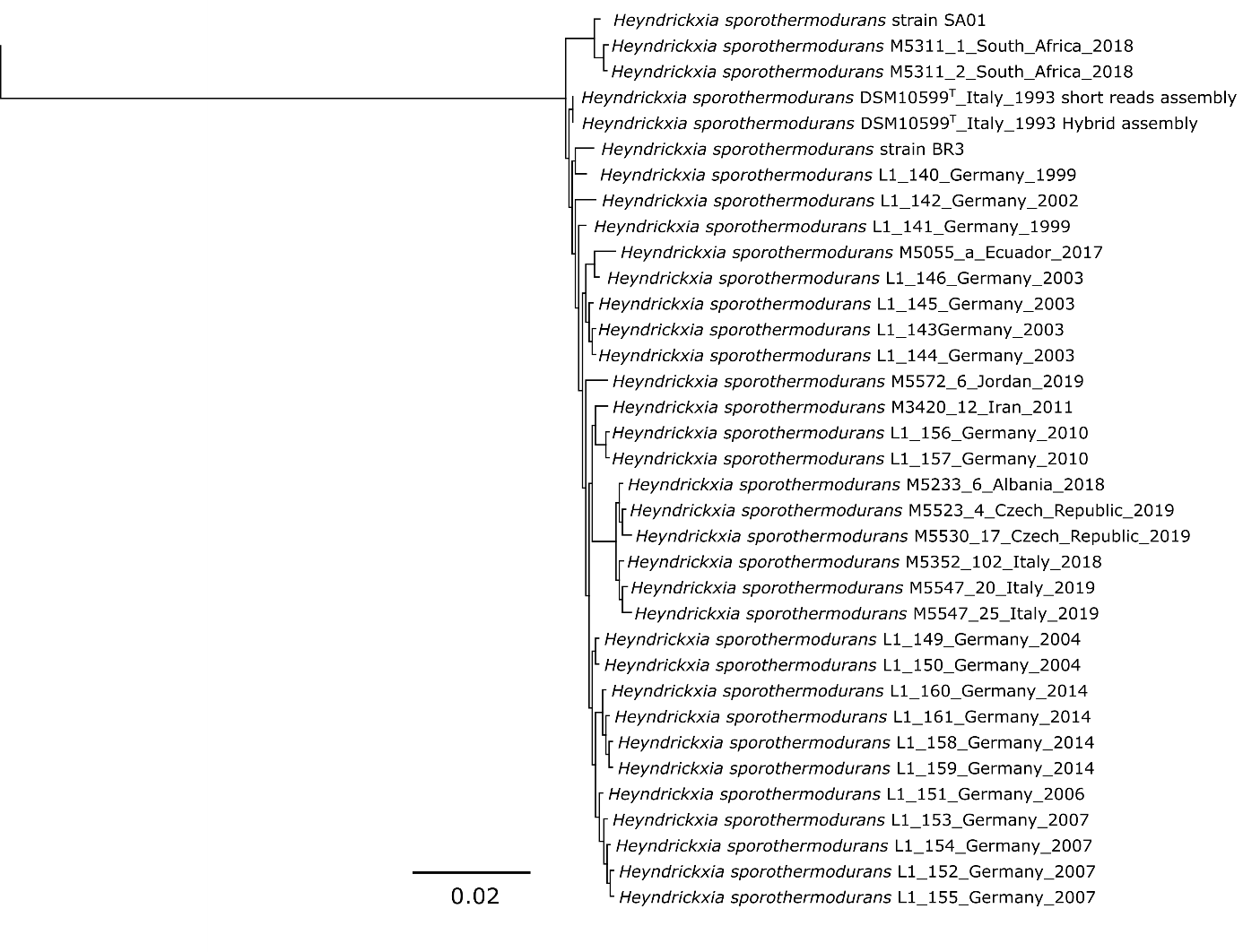


Supplementary Figure 3 Detailed phylogenetic tree analysis of the type strain group of *H. sporothermodurans* strains (n=34) based on single-nucleotide polymorphisms (SNPs). SNPs were called against the type strain as reference at the CSI Phylogeny 1.4 service. The Newick file was processed with Geneious® and InkScape v0.92. The tree was rooted to strain SAD, but only the type strain group are shown in this Figure.

Supplementary Table 3 Single Nucleotide Polymorphism (SNP) matrix of the Heyndrickxia sporothermodurans strains. SNPs were called with type strain DSM 10599^T^ hybrid assembly as reference.

|  | **1** | **2** | **3** | | **4** | **5** | **6** | | **7** | **8** | **9** | | **10** | **11** | **12** | | **13** | **14** | **15** | | **16** | **17** | **18** | | **19** | **20** | **21** | **22** | | **23** | **24** | **25** | | **26** | **27** | **28** | | **29** | **30** | **31** | **32** | **33** | | **34** | **35** | **36** | **37** | |
| --- | --- | --- | --- | --- | --- | --- | --- | --- | --- | --- | --- | --- | --- | --- | --- | --- | --- | --- | --- | --- | --- | --- | --- | --- | --- | --- | --- | --- | --- | --- | --- | --- | --- | --- | --- | --- | --- | --- | --- | --- | --- | --- | --- | --- | --- | --- | --- | --- |
| **1** | 0 | 90 | 75 | | 125 | 120 | 80 | | 93 | 81 | 88 | | 79 | 67 | 81 | | 44 | 47 | 49 | | 60 | 36 | 34 | | 40 | 46 | 45 | 49 | | 47 | 42 | 44 | | 53 | 64 | 53 | | 46 | 44 | 46 | 43 | 4968 | | 77 | 110 | 7927 | 53 | |
| **2** | 90 | 0 | 117 | | 147 | 142 | 122 | | 135 | 123 | 130 | | 121 | 99 | 105 | | 62 | 65 | 67 | | 46 | 76 | 74 | | 80 | 86 | 85 | 89 | | 87 | 88 | 90 | | 75 | 88 | 77 | | 86 | 84 | 86 | 83 | 4992 | | 101 | 132 | 7951 | 75 | |
| **3** | 75 | 117 | 0 | | 152 | 147 | 11 | | 24 | 22 | 29 | | 20 | 94 | 108 | | 71 | 74 | 76 | | 87 | 63 | 61 | | 67 | 73 | 72 | 76 | | 74 | 73 | 75 | | 80 | 91 | 80 | | 73 | 71 | 73 | 70 | 4997 | | 104 | 137 | 7956 | 80 | |
| **4** | 125 | 147 | 152 | | 0 | 11 | 157 | | 170 | 158 | 165 | | 156 | 134 | 118 | | 103 | 106 | 108 | | 119 | 111 | 109 | | 115 | 121 | 120 | 124 | | 122 | 123 | 125 | | 86 | 101 | 98 | | 121 | 119 | 121 | 118 | 5002 | | 114 | 35 | 7962 | 86 | |
| **5** | 120 | 142 | 147 | | 11 | 0 | 152 | | 165 | 153 | 160 | | 151 | 129 | 113 | | 98 | 101 | 103 | | 114 | 106 | 104 | | 110 | 116 | 115 | 119 | | 117 | 118 | 120 | | 81 | 96 | 93 | | 116 | 114 | 116 | 113 | 4997 | | 109 | 30 | 7957 | 81 | |
| **6** | 80 | 122 | 11 | | 157 | 152 | 0 | | 19 | 27 | 34 | | 25 | 99 | 113 | | 76 | 79 | 81 | | 92 | 68 | 66 | | 72 | 78 | 77 | 81 | | 79 | 78 | 80 | | 85 | 96 | 85 | | 78 | 76 | 78 | 75 | 5002 | | 109 | 142 | 7961 | 85 | |
| **7** | 93 | 135 | 24 | | 170 | 165 | 19 | | 0 | 40 | 47 | | 38 | 112 | 126 | | 89 | 92 | 94 | | 105 | 81 | 79 | | 85 | 91 | 90 | 94 | | 92 | 91 | 93 | | 98 | 109 | 98 | | 91 | 89 | 91 | 88 | 5014 | | 122 | 155 | 7973 | 98 | |
| **8** | 81 | 123 | 22 | | 158 | 153 | 27 | | 40 | 0 | 25 | | 18 | 100 | 114 | | 77 | 80 | 82 | | 93 | 69 | 67 | | 73 | 79 | 78 | 82 | | 80 | 79 | 81 | | 86 | 97 | 86 | | 79 | 77 | 79 | 76 | 5003 | | 110 | 143 | 7962 | 86 | |
| **9** | 88 | 130 | 29 | | 165 | 160 | 34 | | 47 | 25 | 0 | | 25 | 107 | 121 | | 84 | 87 | 89 | | 100 | 76 | 74 | | 80 | 86 | 85 | 89 | | 87 | 86 | 88 | | 93 | 104 | 93 | | 86 | 84 | 86 | 83 | 5010 | | 117 | 150 | 7969 | 93 | |
| **10** | 79 | 121 | 20 | | 156 | 151 | 25 | | 38 | 18 | 25 | | 0 | 98 | 112 | | 75 | 78 | 80 | | 91 | 67 | 65 | | 71 | 77 | 76 | 80 | | 78 | 77 | 79 | | 84 | 95 | 84 | | 77 | 75 | 77 | 74 | 5001 | | 108 | 141 | 7960 | 84 | |
| **11** | 67 | 99 | 94 | | 134 | 129 | 99 | | 112 | 100 | 107 | | 98 | 0 | 90 | | 53 | 56 | 58 | | 69 | 53 | 51 | | 57 | 63 | 62 | 66 | | 64 | 65 | 67 | | 62 | 73 | 62 | | 63 | 61 | 63 | 60 | 4977 | | 86 | 119 | 7938 | 62 | |
| **12** | 81 | 105 | 108 | | 118 | 113 | 113 | | 126 | 114 | 121 | | 112 | 90 | 0 | | 59 | 62 | 64 | | 75 | 67 | 65 | | 71 | 77 | 76 | 80 | | 78 | 79 | 81 | | 46 | 57 | 54 | | 77 | 75 | 77 | 74 | 4963 | | 70 | 103 | 7922 | 46 | |
| **13** | 44 | 62 | 71 | | 103 | 98 | 76 | | 89 | 77 | 84 | | 75 | 53 | 59 | | 0 | 5 | 9 | | 32 | 30 | 28 | | 34 | 40 | 39 | 43 | | 41 | 42 | 44 | | 31 | 42 | 31 | | 40 | 38 | 40 | 37 | 4948 | | 55 | 88 | 7907 | 31 | |
| **14** | 47 | 65 | 74 | | 106 | 101 | 79 | | 92 | 80 | 87 | | 78 | 56 | 62 | | 5 | 0 | 12 | | 35 | 33 | 31 | | 37 | 43 | 42 | 46 | | 44 | 45 | 47 | | 34 | 45 | 34 | | 43 | 41 | 43 | 40 | 4951 | | 58 | 91 | 7910 | 34 | |
| **15** | 49 | 67 | 76 | | 108 | 103 | 81 | | 94 | 82 | 89 | | 80 | 58 | 64 | | 9 | 12 | 0 | | 37 | 35 | 33 | | 39 | 45 | 44 | 48 | | 46 | 47 | 49 | | 36 | 47 | 36 | | 45 | 43 | 45 | 42 | 4953 | | 60 | 93 | 7912 | 36 | |
| **16** | 60 | 46 | 87 | | 119 | 114 | 92 | | 105 | 93 | 100 | | 91 | 69 | 75 | | 32 | 35 | 37 | | 0 | 46 | 44 | | 50 | 56 | 55 | 59 | | 57 | 58 | 60 | | 47 | 58 | 47 | | 56 | 54 | 56 | 53 | 4964 | | 71 | 104 | 7923 | 47 | |
| **17** | 36 | 76 | 63 | | 111 | 106 | 68 | | 81 | 69 | 76 | | 67 | 53 | 67 | | 30 | 33 | 35 | | 46 | 0 | 6 | | 20 | 26 | 25 | 29 | | 27 | 34 | 36 | | 39 | 50 | 39 | | 26 | 24 | 26 | 23 | 4956 | | 63 | 96 | 7915 | 39 | |
| **18** | 34 | 74 | 61 | | 109 | 104 | 66 | | 79 | 67 | 74 | | 65 | 51 | 65 | | 28 | 31 | 33 | | 44 | 6 | 0 | | 18 | 24 | 23 | 27 | | 25 | 32 | 34 | | 37 | 48 | 37 | | 24 | 22 | 24 | 21 | 4954 | | 61 | 94 | 7913 | 37 | |
| **19** | 40 | 80 | 67 | | 115 | 110 | 72 | | 85 | 73 | 80 | | 71 | 57 | 71 | | 34 | 37 | 39 | | 50 | 20 | 18 | | 0 | 14 | 13 | 17 | | 15 | 38 | 40 | | 43 | 52 | 43 | | 28 | 26 | 28 | 25 | 4960 | | 67 | 100 | 7919 | 43 | |
| **20** | 46 | 86 | 73 | | 121 | 116 | 78 | | 91 | 79 | 86 | | 77 | 63 | 77 | | 40 | 43 | 45 | | 56 | 26 | 24 | | 14 | 0 | 3 | 7 | | 5 | 44 | 46 | | 49 | 58 | 49 | | 34 | 32 | 34 | 31 | 4966 | | 73 | 106 | 7925 | 49 | |
| **21** | 45 | 85 | 72 | | 120 | 115 | 77 | | 90 | 78 | 85 | | 76 | 62 | 76 | | 39 | 42 | 44 | | 55 | 25 | 23 | | 13 | 3 | 0 | 6 | | 4 | 43 | 45 | | 48 | 57 | 48 | | 33 | 31 | 33 | 30 | 4965 | | 72 | 105 | 7924 | 48 | |
| **22** | 49 | 89 | 76 | | 124 | 119 | 81 | | 94 | 82 | 89 | | 80 | 66 | 80 | | 43 | 46 | 48 | | 59 | 29 | 27 | | 17 | 7 | 6 | 0 | | 8 | 47 | 49 | | 52 | 61 | 52 | | 37 | 35 | 37 | 34 | 4968 | | 76 | 109 | 7928 | 52 | |
| **23** | 47 | 87 | 74 | | 122 | 117 | 79 | | 92 | 80 | 87 | | 78 | 64 | 78 | | 41 | 44 | 46 | | 57 | 27 | 25 | | 15 | 5 | 4 | 8 | | 0 | 45 | 47 | | 50 | 59 | 50 | | 35 | 33 | 35 | 32 | 4967 | | 74 | 107 | 7926 | 50 | |
| **24** | 42 | 88 | 73 | | 123 | 118 | 78 | | 91 | 79 | 86 | | 77 | 65 | 79 | | 42 | 45 | 47 | | 58 | 34 | 32 | | 38 | 44 | 43 | 47 | | 45 | 0 | 4 | | 51 | 62 | 51 | | 44 | 42 | 44 | 41 | 4968 | | 75 | 108 | 7927 | 51 | |
| **25** | 44 | 90 | 75 | | 125 | 120 | 80 | | 93 | 81 | 88 | | 79 | 67 | 81 | | 44 | 47 | 49 | | 60 | 36 | 34 | | 40 | 46 | 45 | 49 | | 47 | 4 | 0 | | 53 | 64 | 53 | | 46 | 44 | 46 | 43 | 4970 | | 77 | 110 | 7929 | 53 | |
| **26** | 53 | 75 | 80 | | 86 | 81 | 85 | | 98 | 86 | 93 | | 84 | 62 | 46 | | 31 | 34 | 36 | | 47 | 39 | 37 | | 43 | 49 | 48 | 52 | | 50 | 51 | 53 | | 0 | 29 | 26 | | 49 | 47 | 49 | 46 | 4931 | | 42 | 71 | 7890 | 0 | |
| **27** | 64 | 88 | 91 | | 101 | 96 | 96 | | 109 | 97 | 104 | | 95 | 73 | 57 | | 42 | 45 | 47 | | 58 | 50 | 48 | | 52 | 58 | 57 | 61 | | 59 | 62 | 64 | | 29 | 0 | 37 | | 58 | 56 | 58 | 55 | 4946 | | 53 | 86 | 7905 | 29 | |
| **28** | 53 | 77 | 80 | | 98 | 93 | 85 | | 98 | 86 | 93 | | 84 | 62 | 54 | | 31 | 34 | 36 | | 47 | 39 | 37 | | 43 | 49 | 48 | 52 | | 50 | 51 | 53 | | 26 | 37 | 0 | | 49 | 47 | 49 | 46 | 4943 | | 50 | 83 | 7902 | 26 | |
| **29** | 46 | 86 | 73 | | 121 | 116 | 78 | | 91 | 79 | 86 | | 77 | 63 | 77 | | 40 | 43 | 45 | | 56 | 26 | 24 | | 28 | 34 | 33 | 37 | | 35 | 44 | 46 | | 49 | 58 | 49 | | 0 | 4 | 8 | 5 | 4966 | | 73 | 106 | 7925 | 49 | |
| **30** | 44 | 84 | 71 | | 119 | 114 | 76 | | 89 | 77 | 84 | | 75 | 61 | 75 | | 38 | 41 | 43 | | 54 | 24 | 22 | | 26 | 32 | 31 | 35 | | 33 | 42 | 44 | | 47 | 56 | 47 | | 4 | 0 | 6 | 3 | 4964 | | 71 | 104 | 7923 | 47 | |
| **31** | 46 | 86 | 73 | | 121 | 116 | 78 | | 91 | 79 | 86 | | 77 | 63 | 77 | | 40 | 43 | 45 | | 56 | 26 | 24 | | 28 | 34 | 33 | 37 | | 35 | 44 | 46 | | 49 | 58 | 49 | | 8 | 6 | 0 | 5 | 4966 | | 73 | 106 | 7925 | 49 | |
| **32** | 43 | 83 | 70 | | 118 | 113 | 75 | | 88 | 76 | 83 | | 74 | 60 | 74 | | 37 | 40 | 42 | | 53 | 23 | 21 | | 25 | 31 | 30 | 34 | | 32 | 41 | 43 | | 46 | 55 | 46 | | 5 | 3 | 5 | 0 | 4963 | | 70 | 103 | 7922 | 46 | |
| **33** | 4968 | 4992 | 4997 | | 5002 | 4997 | 5002 | | 5014 | 5003 | 5010 | | 5001 | 4977 | 4963 | | 4948 | 4951 | 4953 | | 4964 | 4956 | 4954 | | 4960 | 4966 | 4965 | 4968 | | 4967 | 4968 | 4970 | | 4931 | 4946 | 4943 | | 4966 | 4964 | 4966 | 4963 | 0 | | 4959 | 4987 | 7950 | 4931 | |
| **34** | 77 | 101 | 104 | | 114 | 109 | 109 | | 122 | 110 | 117 | | 108 | 86 | 70 | | 55 | 58 | 60 | | 71 | 63 | 61 | | 67 | 73 | 72 | 76 | | 74 | 75 | 77 | | 42 | 53 | 50 | | 73 | 71 | 73 | 70 | 4959 | | 0 | 99 | 7918 | 42 | |
| **35** | 110 | 132 | 137 | | 35 | 30 | 142 | | 155 | 143 | 150 | | 141 | 119 | 103 | | 88 | 91 | 93 | | 104 | 96 | 94 | | 100 | 106 | 105 | 109 | | 107 | 108 | 110 | | 71 | 86 | 83 | | 106 | 104 | 106 | 103 | 4987 | | 99 | 0 | 7947 | 71 | |
| **36** | 7927 | 7951 | 7956 | | 7962 | 7957 | 7961 | | 7973 | 7962 | 7969 | | 7960 | 7938 | 7922 | | 7907 | 7910 | 7912 | | 7923 | 7915 | 7913 | | 7919 | 7925 | 7924 | 7928 | | 7926 | 7927 | 7929 | | 7890 | 7905 | 7902 | | 7925 | 7923 | 7925 | 7922 | 7950 | | 7918 | 7947 | 0 | 7890 | |
| **37** | 53 | 75 | 80 | | 86 | 81 | 85 | | 98 | 86 | 93 | | 84 | 62 | 46 | | 31 | 34 | 36 | | 47 | 39 | 37 | | 43 | 49 | 48 | 52 | | 50 | 51 | 53 | | 0 | 29 | 26 | | 49 | 47 | 49 | 46 | 4931 | | 42 | 71 | 7890 | 0 | |
| min: 0 max: 7973 | | |  | |  |  |  | |  |  |  | |  |  |  | |  |  |  | |  |  |  | |  |  |  |  | |  |  |  | |  |  |  | |  |  |  |  |  | |  |  |  |  | |
|  | | | |  | | | |  | | | |  | | | |  | | | |  | | | |  | | | | |  | | | |  | | | |  | | | | | |  | | | | |  |
| SNPs Matrix decoding table | | | | | | | | | | | | | | | | | | | | | | | | | | | | | | | | | | | | | | | | | | | | | | | |  |
| Strain | | | | Code | | | |  | | | | Strain | | | | Code | | | |  | | | | Strain | | | | | Code | | | |  | | | | Strain | | | | | | Code | | | | |  |
| M3420-12 | | | | 1 | | | |  |  |  |  | M5572-6 | | | | 11 | | | |  |  |  |  | L1_153 | | | | | 21 | | | |  |  |  |  | L1_160 | | | | | | 31 | | | | |  |
| M5055-A | | | | 2 | | | |  |  |  |  | L1_142 | | | | 12 | | | |  |  |  |  | L1_154 | | | | | 22 | | | |  |  |  |  | L1_161 | | | | | | 32 | | | | |  |
| M5233-6 | | | | 3 | | | |  |  |  |  | L1_143 | | | | 13 | | | |  |  |  |  | L1_155 | | | | | 23 | | | |  |  |  |  | B4102 | | | | | | 33 | | | | |  |
| M5311-1 | | | | 4 | | | |  |  |  |  | L1_144 | | | | 14 | | | |  |  |  |  | L1_156 | | | | | 24 | | | |  |  |  |  | BR3_SRR8741693 | | | | | | 34 | | | | |  |
| M5311-2 | | | | 5 | | | |  |  |  |  | L1_145 | | | | 15 | | | |  |  |  |  | L1_157 | | | | | 25 | | | |  |  |  |  | SA01_SRR8732969 | | | | | | 35 | | | | |  |
| M5523-4 | | | | 6 | | | |  |  |  |  | L1_146 | | | | 16 | | | |  |  |  |  | DSM 10599^T *^ | | | | | 26 | | | |  |  |  |  | SAD_SRR8732968 | | | | | | 36 | | | | |  |
| M5530-17 | | | | 7 | | | |  |  |  |  | L1_149 | | | | 17 | | | |  |  |  |  | L1_140 | | | | | 27 | | | |  |  |  |  | Reference/Hybrid assembly | | | | | | 37 | | | | |  |
| M5547-20 | | | | 8 | | | |  |  |  |  | L1_150 | | | | 18 | | | |  |  |  |  | L1_141 | | | | | 28 | | | |  |  |  |  | *short read assembly | | | | | | | | | | |  |
| M5547-25 | | | | 9 | | | |  |  |  |  | L1_151 | | | | 19 | | | |  |  |  |  | L1_158 | | | | | 29 | | | |  |  |  |  |  |  |  |  |  |  |  |  |  |  |  |  |
| M5352-102 | | | | 10 | | | |  |  |  |  | L1_152 | | | | 20 | | | |  |  |  |  | L1_159 | | | | | 30 | | | |  |  |  |  |  |  |  |  |  |  |  |  |  |  |  |  |
